# A Trial of Three Rounds of Mass Drug Administration with Azithromycin for Yaws

**DOI:** 10.1056/NEJMoa2109449

**Published:** 2022-01-06

**Authors:** Lucy N. John, Camila G. Beiras, Wendy Houinei, Monica Medappa, Maria Sabok, Reman Kolmau, Eunice Jonathan, Edward Maika, James K. Wangi, Petra Pospíšilová, David Šmajs, Dan Ouchi, Iván Galván-Femenía, Mathew A Beale, Lorenzo Giacani, Bonaventura Clotet, Eric Q. Mooring, Michael Marks, Marti Vall-Mayans, Oriol Mitjà

**Affiliations:** 1 National Department of Health, Aopi Centre, Port Moresby, Papua New Guinea; 2 Infectious Diseases Department and Fight AIDS and Infectious Diseases Foundation, Hospital Universitari Germans Trias i Pujol, Badalona, Catalonia (Spain); 3 School of Medicine and Health Sciences, University of Papua New Guinea, Port Moresby, Papua New Guinea; 4 Barcelona Institute for Global Health -Faculty of Medicine, University of Barcelona, Barcelona, Spain; 5 Department of Biology, Faculty of Medicine, Masaryk University, Brno, Czech Republic; 6 New Ireland Provincial Health Authority, Kavieng, Papua New Guinea; 7 Lihir Medical Centre, International SOS, Lihir Island, Papua New Guinea; 8 WHO Representative Office in Papua New Guinea, Port Moresby, Papua New Guinea; 9 Genomes for Life-GCAT lab Group, Institute for Health Science Research Germans Trias i Pujol, Badalona, Spain; 10 Institute for Research in Biomedicine, Barcelona Institute of Science and Technology, Barcelona, Spain; 11 Parasites and Microbes Programme, Wellcome Sanger Institute, Hinxton, United Kingdom; 12 Department of Medicine, Division of Allergy and Infectious Diseases, and Department of Global Health, University of Washington, Seattle, WA, USA; 13 IrsiCaixa AIDS Research Institute, Badalona, Spain; 14 Universitat de Vic - Universitat Central de Catalunya, Vic, Spain; 15 Department of Epidemiology, Harvard T.H. Chan School of Public Health, Boston, Massachusetts, USA; 16 Clinical Research Department, London School of Hygiene and Tropical Medicine, London, United Kingdom; 17 Hospital for Tropical Diseases, University College London Hospitals, London, United Kingdom

## Abstract

**Background:**

*Treponema pallidum* subsp. *pertenue* causes yaws. Strategies to better control and hopefully eliminate yaws are needed.

**Methods:**

We conducted an open-label cluster-randomized community trial in a yaws-endemic area of Papua New Guinea. Thirty-eight wards were randomized to receive either one mass drug administration (MDA) round followed by two target treatment of active cases rounds (control arm) or three MDA rounds (experimental arm) at 6-month intervals. The difference in the prevalence of active and latent yaws were measured at 18-month surveys.

**Results:**

Nineteen wards (30,438 individuals) were randomized to the control arm and 19 (26,238 individuals) to the experimental arm. 24,848 azithromycin doses were administered in the control arm (22,033 at baseline, 207 participants with yaws-like lesions and 2,608 contacts at 6-month and 12-month), compared to 59,852 doses in the experimental arm. At 18 months, the prevalence of active yaws had decreased from 0.46% (102/22,033) to 0.16% (47/29,954) in the control arm and from 0.43% (87/20,331) to 0.04% (10/25,987) in the experimental arm (RR 3.54; 95%CI 1.72–7.27). The prevalence of other infectious ulcers decreased to a similar extent in the two study arms. The prevalence of latent yaws at 18 months, assessed in 994 and 945 children in the control and experimental arms, was 6.54% (5.00–8.08) and 3.28% (2.14–4.42), respectively (RR 2.03; 1.12–3.7). Three cases with resistance to macrolides were found in the experimental arm.

**Conclusions:**

These data show that three rounds of azithromycin MDA 6 months apart are better than one round of azithromycin MDA with two rounds of targeted treatment for decreasing the community prevalence of yaws. Monitoring for the emergence and spread of antimicrobial resistance is needed. (ClinicalTrials.gov number, NCT03490123.)

Yaws, an infection caused by *Treponema pallidum* subsp*. pertenue*, affects the skin and long bones of children in poor rural communities in the tropics.^1^ In the absence of an eradication campaign, yaws would be estimated to cause 1.6 million disability-adjusted life-years lost between 2015–2050.^2^

The finding that a single oral dose of azithromycin is as effective as penicillin G benzathine for treating the disease^3^ prompted WHO to launch a worldwide program to eradicate yaws by 2030.^4,5^ The current principle of yaws eradication (the Morges strategy) is based on a single round of mass drug administration (MDA) with azithromycin to the entire community, followed by treatment of active cases and their contacts every six months called total targeted treatment (TTT).^4,5^ As of August 2021, WHO in collaboration with a Brazilian pharmaceutical company, has provided >10 million tablets to support MDA in several countries, including Cameroon, Central African Republic, Congo, Ivory Coast, Ghana, Liberia, Indonesia, Papua New Guinea (PNG), the Solomon Islands and Vanuatu.^6^

Studies conducted in PNG, the Solomon Islands, and Ghana have all shown that one round of MDA will not suffice to stop transmission of infection,^7–9^ presumably due to the relapse of untreated latent infections amongst individuals who had not been treated during MDA.^10^ Mathematical modeling data show that a high coverage of latent cases is required during treatment campaigns to have a high likelihood of achieving eradication.^11^ However, targeted treatment, which focuses only on active infection cases and contacts, may not achieve a sufficient level of treatment coverage amongst latently infected individuals.^12^

Based on both empirical and modeling data, ^13,14^ we hypothesized that three rounds of MDA with azithromycin before switching to targeted treatment would be more effective than standard care with one round of MDA for reducing the prevalence of active and latent yaws.

## Methods

### Study setting and participants

From April 2018 through October 2019, we conducted a cluster-randomized open-label phase 3 community trial in the Namatanai District of the New Ireland Province of PNG. The district consists of six local level government areas, three of which were selected for this study: Matalai Rural, Namatanai Rural, and Sentral Niu Ailan Rural. The estimated resident population of the three local level government areas amounted to 56,676 people in 38 wards based on a census conducted in 2016 with correction for population growth.^15^ Wards are the lowest administrative unit encompassing a group of 3 to 5 villages that share the same school and/or church. For the purpose of the study, clusters were individuals living in the same ward. We randomized 38 wards to either an experimental or a control arm. All individuals older than one month and living in the study wards were eligible to participate. Exclusion criteria for receiving azithromycin were known allergy to macrolide antibiotics, severely ill patients, and refusal.

Thirty-eight field teams (i.e., one in each ward) were responsible for delivering the treatment intervention and collecting study data. Field teams were selected by the PNG National Department of Health and consisted of a team leader (either a community health worker or a nurse) and four community health volunteers. Village leaders from the study wards were engaged to serve as gatekeepers for entry into communities and ensure participant recruitment and retention. Villagers were informed in advance about the date of the study visit and were invited to attend a central point in the village by means of “Toksave” (public notice in Tok Pisin, the most widely spoken language in PNG).

All study participants — or their parent or guardian when children — provided oral informed consent for screening and treatment. Individuals with suspected skin lesions of yaws provided written informed consent for collecting biological samples. The study protocol was approved by the Medical Research Advisory Committee of the PNG National Department of Health (MRAC No: 17.19), which authorized oral consent.

### Randomization and interventions

Randomization was performed at the ward level (1:1 allocation to either the control or experimental arm) and stratified by local level government using a computer-generated random allocation sequence. The control arm received the Morges strategy (i.e., a single MDA with azithromycin at baseline, followed by two rounds of targeted treatment at 6 and 12 months), whereas the experimental arm received three rounds of MDA with azithromycin at baseline, 6, and 12 months. MDA consisted of blanket treatment irrespective of infection status. Targeted treatment consisted of pro-active screening of all participants for evidence of yaws; individuals with yaws-like lesions, and their household contacts or school classmates were offered treatment.

Azithromycin (Kern Pharma) was administered as a single dose orally, under direct observation, according to weight-to-age correspondence recommended by the WHO guidelines: age ≥15 (4 tablets of 500 mg), age 10-14 (3 tablets), age 5-9 (2 tablets), age 1-4 years (1 tablet or 7.5mL of azithromycin syrup). Azithromycin syrup (5mL) was offered to children aged 1-12 months. Treatment was donated by Kern Pharma (Barcelona, Spain) and provided without cost to study participants.

The implementation of each treatment round, either MDA or targeted treatment, lasted five days. On the first three days, villagers were invited to attend a central point for assessment and treatment. On the subsequent two days, the field teams conducted house-to-house visits to find residents who had not been treated at the central points (mop-up exercise). Census and tally sheets of the population surveyed were used to assess coverage.

### Outcomes and procedures

The co-primary outcomes were the prevalence of PCR-confirmed active yaws in the entire trial population and the prevalence of serologically confirmed latent yaws measured in a subgroup of asymptomatic children aged 1-15 years, at 18-month surveys. We also measured the prevalence of active yaws at baseline, 6-month, and 12-month surveys. Secondary outcomes included the genetic diversity of yaws, the proportion of macrolide-resistant yaws strains, and the prevalence of ulcers caused by *Haemophilus ducreyi* which coexists with yaws as a cause of skin ulcers and responds well to azithromycin.^16^

Clinical surveys for active yaws prevalence were undertaken in the entire resident population. The clinical definition of active yaws was an ulcerative or nodular skin lesion of more than 2 cm in diameter (the size of 5 toea PNG coin). A lesion sample swab was obtained for each patient and shipped to Masaryk University (Czech Republic) for detection of *T*. *p*. *pertenue* DNA, strain genotyping, identification of mutations associated with resistance to azithromycin, and detection of *H*. *ducreyi* DNA. Methods for clinic-demographic data collection and laboratory analyses are explained in the Supplementary appendix. Briefly, PCR was performed using a nested-PCR protocol.^17^ Allelic profiles based on sequences of TP0548, TP0488, and TP0858 genes (Table S1) were assigned to individual samples and used to determine *T*. *p*. *pertenue* Mean Evolutionary Diversity at each timepoint, the presence of point mutations in the 23S rRNA genes causing macrolide resistance (A2058G and A2059G) were identified by sequencing, and all samples were screened by qPCR targeting *H*. *ducreyi* as described previously.^18^ Laboratory personnel who carried out the assessment were blinded to the treatment allocation.

The prevalence of latent yaws was assessed in 12 randomly selected clusters (i.e., wards) out of 19 wards from each treatment arm at 18 months. In each cluster, we randomly selected a sample of 75 asymptomatic children aged 1-15 years, totaling 900 children per arm. Latent yaws was assessed by finger prick rapid quantitative serological test (dual-path platform syphilis screen and confirm assay; Chembio Diagnostics, Medford, NY, USA).^19^ A positive yaws serology was considered if the optical density values of the treponemal and non-treponemal lines were ≥12 and ≥30, respectively.^20^ High-titer serology was considered if the density of the non-treponemal line was ≥90.

### Sample size considerations and statistical analysis

Based on a previous public health intervention for yaws elimination,^7^ we expected a 0.11% prevalence of PCR-confirmed active yaws in the control arm at 18 months and a 0.75 coefficient of variation of the ward-level prevalence. The proposed study area is divided into 38 wards (19 wards per treatment arm); the harmonic mean of the estimated population per ward is 1,177, and we expected that 80% of the population would be screened during clinical surveys. Based on these assumptions, we would have 77.3% power to detect an 80% reduction in the prevalence of PCR-confirmed active yaws in the experimental arm compared to the control arm at a two-sided 0.05 alpha significance level.

For latent yaws, we expected a 6% prevalence in children in the control arm, and a 0.30 coefficient of variation of the ward-level prevalence. Based on these assumptions, 1,800 individuals would provide 80% power to detect a 50% reduction in the prevalence of latent yaws using a significance threshold of a two-sided alpha value of 0.05.

The primary analysis included all individuals who resided in the study wards, met the selection criteria, and were present for the 18-month clinical survey. Prevalence with two-sided confidence intervals (CI) was calculated using the Wald method.^21^ For the co-primary endpoints, we had originally planned to estimate odds ratios, but in adherence to the journal guidelines we used relative risks, instead, because it is less likely to overestimate risks and easier to interpret. Finally, we fitted a generalized estimating equation (GEE) log-binomial model that accounted for clustering^22^ to estimate a relative risk (RR) comparing the prevalence of PCR-confirmed active yaws among individuals in the experimental arm and in the control arm. To determine whether the log-RR of PCR-confirmed yaws between arms was significantly different from zero at a two-sided alpha level of 0.05, we used a Wald test on the robust standard error from the fitted treatment effect coefficient. We used the same methodology for the latent yaws endpoint. We did not adjust the type I error for multiplicity because we considered that both co-primary endpoints individually must show statistically significant treatment benefit. Coverage rates were calculated using tally sheets of the population surveyed in each treatment round (numerator), and estimates of the eligible population based on the 2016 census adjusted for population growth (denominator).^15^ We applied a 5% yearly growth factor to obtain the 2018-corrected population. Treatment arm allocation was blinded to the statisticians, who were allowed access only to coded groups. All analyses were performed in R software (v3.6.3).

## Results

### Study population and coverage

**Figure 1:**
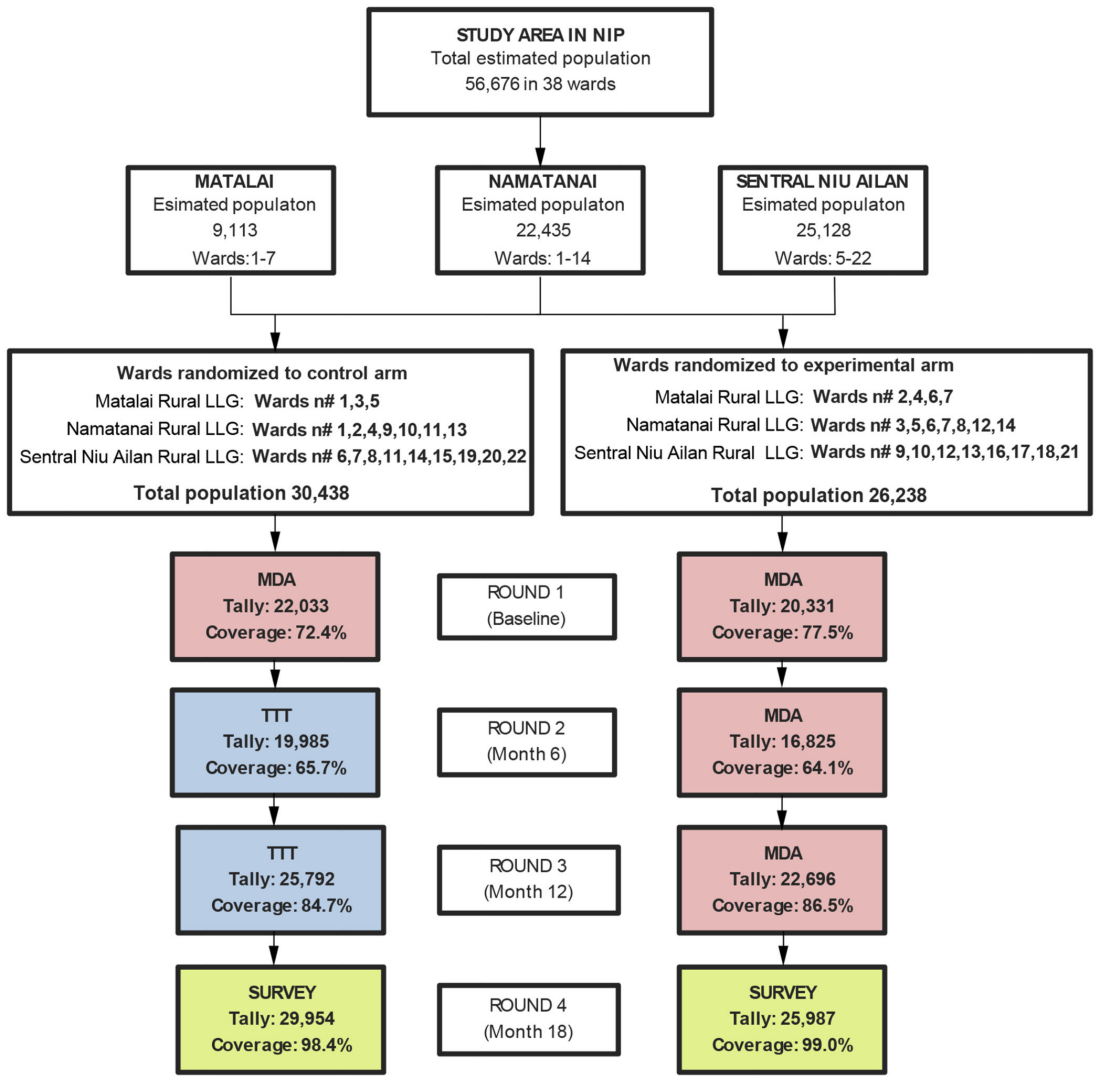
Trial profile **MDA:** Mass drug administration. **TTT:** total targeted treatment. The estimated population was based on a survey conducted in 2016 for bed net distribution. The study area consisted of three Local Level Government (LLG) areas in Namatanai District. Each LLG in the study area has between 7-22 wards that are identified with consecutive numbers. Wards are the lowest administrative unit encompassing a group of 3 to 5 villages that share the same school and/or church. We used the ward as the randomization unit to reduce the risk of spill over between treatment arms; ward residents often attend the same school or church, but this is less common among residents of different wards. The mean population of the wards in Namatanai is 1,177 individuals (range 679-1,902), except for the district capital ward n# 10 in the Namatanai Rural LLG with 3,578. We selected 7/7 wards in Matalai Rural LLG, 14/14 wards in Namatanai Rural LLG, 17/22 wards in Sentral Niu Ailan LLG. Five island wards of Sentral Niu Ailand (1-4) were excluded due to remoteness. The coverage at the 6-month survey (Oct 2018) was lower due to a tropical cyclone that caused roadblocks and reduced access to many villages.

The study population consisted of 56,676 people living in 38 wards that were randomly assigned to either the control arm (19 wards, 30,438 individuals) or the experimental arm (19 wards, 26,238 individuals) (Figure 1). Baseline age and gender distribution were similar between study arms (Table 1). A total of 42,362 (74.7%), 36,810 (64.9%), 48,488 (85.6%) individuals received the intervention – MDA or targeted treatment – at baseline, 6 months, and 12 months, respectively. Coverage did not differ between the two treatment arms at each time point (Figure 1). The overall number of doses of azithromycin given was 24,848 in the control arm (22,033 at baseline, 207 participants with yaws-like lesions and 2,608 contacts at 6-month and 12-months targeted treatment rounds), compared to 59,852 in the experimental arm. We could locate and assess 55,941 (98.7%) individuals for the primary outcome assessment at 18 months.

### Changes in prevalence of active yaws

During the study period, we identified 1,026 ulcers, of which 297 were PCR-confirmed as yaws (Table 2). At baseline, the prevalence of active yaws was 0.46% (102/22,033) in the control arm and 0.43% (87/20,331) in the experimental arm. At the 18-month survey, the prevalence of active yaws decreased to 0.16% (47/29,954) and 0.04% (10/25,987) in the control and experimental arms, respectively (RR 3.54; 95%CI 1.72 – 7.27). The prevalence of active yaws was highest in children younger than 15 years (Figure S2) and the rebound in the control arm was particularly marked amongst the 6 to 10 years age group.

**Figure 2:**
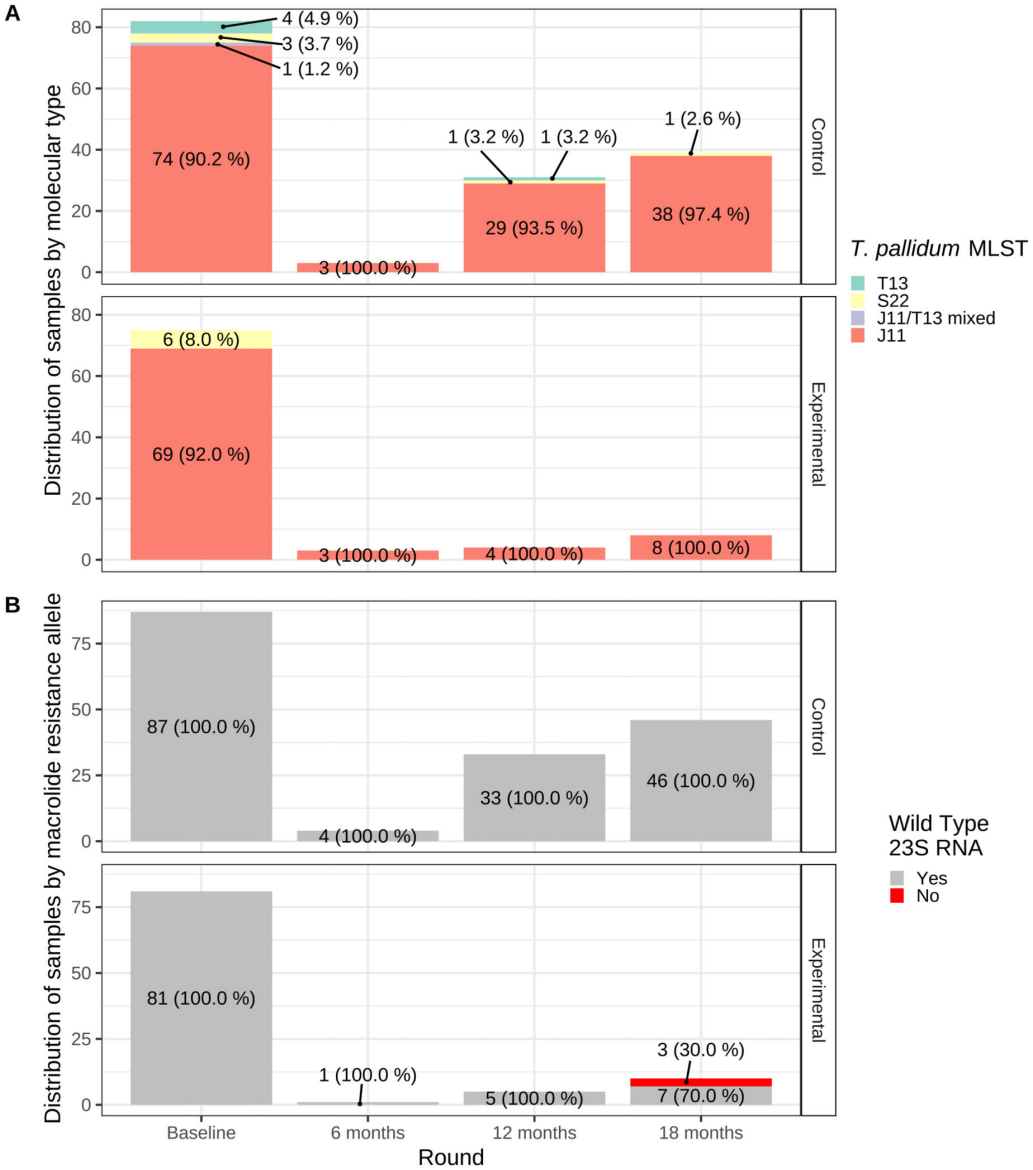
Allelic profile and macrolide resistance in PCR-confirmed yaws ulcer episodes. We applied a variation of the three-amplicon multi-locus sequence typing (MLST) scheme previously described for *T. pallidum subs. pertenue^23^* (see Supplementary Methods for details) which allowed us to grouping bacterial strains according to genetic relatedness, leading to three allelic profiles: J11, S22, and T13. TPE evolutionary distance (K80/K2P model) for the different rounds (not separated by arm) was 0.00207 at baseline, <0.0001 at 6 month, 0.00151, at 12 month, and 0.00055 at 18 months (P-value for the AMOVA when comparing baseline to 18 month 0. 2248)

Figure 2 shows molecular biology findings of PCR-confirmed yaws ulcers sampled in each survey (additional clinic and serologic findings are provided in Table S3). Using multi-locus sequence typing (MLST), three different allelic profiles were identified: J11, S22, and T13 (corresponding to JG8, SE7, and TD6 described previously).^23^ The proportion of cases with the MLST profile J11 was 91.7% (144/157) at baseline; other types identified were S22, 5.7% (9/157), and T13, 3.2% (5/157). Molecular diversity decreased over time with strain J11 causing 100% (6/6), 94.3% (33/35), and 97.9% (46/47) of cases at 6-, 12-, and 18-month surveys, respectively. All yaws cases had a wild type 23S RNA, except three cases at the 18-month survey in the experimental arm, which had the A2058G mutation associated with macrolide resistance.

### Other ulcerative pathogens

Of the 729 non-yaws ulcers, 271 (37.2%) had an *H*. *ducreyi* PCR-positive result and 458 (62.8%) were non-*T*. *p*. *pertenue*, non-*H*. *ducreyi* ulcers. At baseline, the prevalence of ulcers caused by *H*. *ducreyi* was 0.24% (0.17 – 0.30) in the control arm and 0.33% (0.25 – 0.41) in the experimental arm. The prevalence dropped in the 6-month survey and returned to values similar to those of the baseline in both study arms at 18 months (Table 2). The first MDA with azithromycin had a mild impact on ulcers with non- *T*. *p*. *pertenue* non-*H*. *ducreyi* ulcers, and the 18-month prevalence was similar to baseline in both study arms.

### Changes in the prevalence of latent yaws

The serosurvey for latent yaws at 18 months was conducted in 994 and 945 children in the control and experimental arms, respectively (Table 3). The prevalence of dual treponemal and non-treponemal reactivity, measured using the dual-path platform optical density reader, was 6.54% (95%CI 5.00 – 8.08) and 3.28% (2.14 – 4.42) in the control and experimental arms, respectively (adjusted RR 2.03; 95%CI 1.12 – 3.70). The prevalence of high-titer serology (optical density ≥90) was 1.41% (0.68 – 2.14) and 0.53% (0.07 – 0.99) in the control and experimental arms, respectively (aRR 2.58; 95%CI 0.92 – 7.22).

## Discussion

In this cluster-randomized trial, we found a reduction of active yaws prevalence after one round of MDA with azithromycin in line with previous mass treatment interventions^7–9^. In clusters that received two additional MDA rounds, prevalence remained low at 18 months compared to those that received only a single round of MDA followed by targeted treatment rounds. This finding is supported by a lower seroprevalence of infection amongst asymptomatic children in the experimental arm compared to the control arm, consistent with a reduction in the burden of latent yaws. As anticipated, substantially more individuals were treated with azithromycin in the experimental arm. Over the 18-month period 500 extra doses of azithromycin were administered for each case of active yaws prevented and approximately 100 extra doses per case of latent yaws in children avoided.

Three consecutive MDA rounds reduced both active and latent yaws but did not get all the way to elimination. This result could be because cases spilled over from the wards in the control to the experimental arm; not enough latent cases were captured; or active cases were missed. Assessing this is hard since we did not have longitudinal information at the individual level of the whole population. In addition, we found the A2058G mutation suggesting macrolide resistant-strains of *T*. *p*. *pertenue* in 3 participants in the MDA arm. The frequency of resistance was similar to that we observed after repeated targeted treatment rounds in the nearby island of Lihir. ^10,24^

Similar to other PCR-based studies of ulcerative skin lesions in areas with a high yaws incidence,^25,26^ we found a high proportion of ulcers caused by microorganisms other than *T*. *p*. *pertenue*. Patients with ulcers caused by *H*. *ducreyi* also benefited from the initial MDA with azithromycin, but the prevalence of these ulcers rebounded in subsequent rounds. The overall community prevalence of ulcers of unknown etiology was unchanged following mass azithromycin treatment, underscoring the importance of non-pharmacological strategies (e.g., hygiene and sanitation) for the epidemiological control of skin ulcers.^27^

Our study has several strengths compared to previous studies. First, the cluster-randomized trial design provides evidence that the decrease in the prevalence of both active and latent yaws is attributable to the experimental intervention. Second, the generalizability of our findings from a subdistrict located in a large landmass with contiguous non-treated subdistricts is higher compared to previous efforts in smaller islands. In addition, the intervention was performed within the structures of the routine health system with local resources at the community level and lead by the National Department of Health. There are several limitations to our study including the absence of an updated population census. Furthermore, we cannot comment on whether a different number of MDA rounds might have been more effective. It would have been helpful in this regard to see if counts stayed low in the experimental arm beyond 6 months after the last MDA, but this could not be done due to Covid-19 travel restrictions. In addition, we did not assess for the emergence of macrolide resistance in other organisms.

Our data suggest that the strategy for yaws eradication should consider additional MDA rounds. The selection and spread of antimicrobial resistance and the failure to eliminate yaws in the area after three consecutive MDA rounds highlight the need to maintain careful clinical and molecular surveillance for the emergence of antimicrobial resistance in *T*. *p*. *pertenue* and other bacterial organisms associated with MDA use.^13^

## Supplementary Material

Supplement

## Figures and Tables

**Table 1:** Characteristics of participants at baseline

	Total (n=42364)	Control arm (n=22033)	Experimental arm (n=20331)
**Local level government**			
Matalai	6810	2813 (12.8%)	3997 (19.7%)
Namatanai	16667	8148 (37%)	8519 (41.9%)
Sentral Niu Ailan	18887	11072 (50.3%)	7815 (38.4%)
**Gender**			
Female	20311	10556 (47.9%)	9755 (48.0%)
Male	22053	11477 (52.1%)	10576 (52.0%)
**Age group**			
≤5y	6997	3604 (16.4%)	3393 (16.7%)
6-10y	5348	2763 (12.5%)	2585 (12.7%)
11-15y	6075	3209 (14.6%)	2866 (14.1%)
>15y	23944	12457 (56.5%)	11487 (56.5%)

**Table 2:** Prevalence of ulcers with *Treponema pallidum pertenue* DNA and *Haemophilus ducreyi* DNA detected by PCR, and non-Tp non-Hd ulcers

	Control arm		Experimental arm		Relative risk (95%CI)*	p-value
	n	Prevalence % (95%CI)	n	Prevalence % (95%CI)		
***T*. *p*. *pertenue***						
Baseline	102	0.46 (0.38-0.55)	87	0.43 (0.34-0.52)	1.08 (0.81 - 1.44)	<0.001
6 months	5	0.03 (0.01-0.05)	3	0.02 (0.00-0.04)	1.40 (0.34 - 5.87)	
12 months	38	0.15 (0.10-0.20)	5	0.02 (0.00-0.04)	6.69 (2.63 - 16.99)	
18months	47	0.16 (0.11-0.20)	10	0.04 (0.02-0.07)	3.54 (1.72 - 7.27)	
***H*. *ducreyi***						
Baseline	52	0.24 (0.17-0.30)	67	0.33 (0.25-0.41)	0.72 (0.50 - 1.03)	
6 months	7	0.04 (0.01-0.07)	1	0.01 (0.00-0.02)	5.89 (0.73 - 47.89)	
12 months	54	0.21 (0.16-0.27)	17	0.07 (0.04-0.11)	2.80 (1.62 - 4.82)	
18months	40	0.13 (0.09-0.18)	33	0.13 (0.08-0.17)	0.91 (0.6 - 1.39)	
**Non-Tp non-Hd ulcers**						
Baseline	79	0.36 (0.28-0.44)	84	0.41 (0.33-0.50)	0.87 (0.64 - 1.18)	
6 months	43	0.22 (0.16-0.28)	44	0.26 (0.19-0.34)	0.82 (0.54 - 1.25)	
12 months	60	0.23 (0.17-0.29)	8	0.04 (0.01-0.06)	6.60 (3.16 - 13.8)	
18months	67	0.22 (0.17-0.28)	73	0.28 (0.22-0.35)	0.69 (0.53 - 0.9)	

* We applied a log-binomial model to estimate the Relative Risk (adjusted for cluster). P-value is presented for the co-primary outcome *T*. *p*. *pertenue* at 18 months.

**CI:** confidence interval. **Non-Tp non-Hd:** Non-*T*. *p*. *pertenue* non-*H*. *ducrey* *i*

**Table 3:** Prevalence of latent yaws at 18 months ^a^

	Control arm (N=994)		Experimental arm (N=945)		Relative Risk (95%CI)*	adjusted RR using Age* (95%CI)	p-value
	n	% 95CI	n	% 95CI			
**Reactive in treponemal line** (T-line ≥12)	433	43.56 (40.48 - 46.64)	322	34.07 (31.05 - 37.10)	1.29 (0.86 - 1.93)	1.31 (0.88 - 1.97)	0.020
**Positive serology** (T-line ≥12 and NT-line ≥30)	65	6.54 (5.00 - 8.08)	31	3.28 (2.14 - 4.42)	1.99 (1.10 - 3.61)	2.03 (1.12 - 3.7)	
**High-titer serology** (T-line ≥12 and NT-line ≥90)	14	1.41 (0.68 - 2.14)	5	0.53 (0.07 - 0.99)	2.66 (0.98 - 7.23)	2.58 (0.92 - 7.22)	

* We applied a log-binomial model to estimate Relative Risk (adjusted for cluster). P-value is presented for the co-primary outcome positive serology.

**T:** treponemal. **NT:** non-treponemal. **CI:** confidence interval. **RR:** relative risk (adjusted for cluster).

a Assessed by dual path platform syphilis screen and confirm assay on a finger capillary blood. NT line density thresholds ≥ 30 and ≥ 90 corresponds to rapid plasma reagin 1:4, and 1:16, respectively.^20^ A participant was considered to have positive yaws serology if the T-line ≥12 and NT-line ≥30, and to have a high-titer serology if the T-line ≥12 and NT-line ≥90.
