# Supplementary material for: A Trial of Three Rounds of Mass Drug Administration with Azithromycin for Yaws
Source: N Engl J Med. Author manuscript; Available in PMC 2022 Jan 11. (PMC7612200; doi:10.1056/NEJMoa2109449)
Supplement: Supplement [file EMS140575-supplement-Supplement.pdf]

Supplementary Appendix

Table of contents

ADDITIONAL METHODS........ 2

Setting........ 2

Clinical surveys for active yaws 2

Molecular characterization of lesion swab samples........ 2

Latent yaws surveys 3

Table S1. Primers used for the nested-PCR amplification of typing loci and the 23S rRNA gene of *T. p. pertenue.* 4

ADDITIONAL RESULTS 5

Table S2. Characteristics of participants at baseline, 6-, 12-, and 18-month surveys. 5

Figure S1. Prevalence of ulcers from each source over time......... 6

Figure S2. Change in the prevalence by age of (A) *Treponema pallidum pertenue* ulcers; (B) *Haemophilus ducreyi* ulcers, and (C) non-*T. p. pertenue* non- *H. ducreyi* ulcers. 7

Table S3. Clinical and laboratory characteristics of PCR-confirmed yaws ulcer episodes......... 8

Table S4. Characteristics of individuals included in the serosurvey of latent yaws at 18 months......... 9

REFERENCES........ 10

ADDITIONAL METHODS

Setting

Before study commencement, teams underwent standardized training in clinical diagnosis of yaws, rapid diagnostic testing, collection of lesion samples, management of adverse events, and completion of the documentation in line with the Good Clinical Practice guidelines. Each team was equipped with a box containing all material required for the trial and mobile phones for communication with study supervisors in the field.

Clinical surveys for active yaws

Clinical surveys for active yaws prevalence were undertaken in the entire resident population. The clinical definition of active yaws was an ulcerative or nodular skin lesion of more than 1 2 cm in diameter (the size of 5 toea PNG coin). Demographic and clinical data were collected for every case of suspected yaws, including age, gender, number of ulcers, size and duration, and travel history in the preceding 6 months.

Molecular characterization of lesion swab samples

Swab samples were stored in a transport medium (100mM TRIS, 100mM EDTA, 1% SDS) at -20 °C until DNA isolation. DNA was isolated using QIAamp DNA Blood Mini Kit (Qiagen) directly from transport medium according to manufacturer´s instructions and eluted into 100 microliters of AE buffer (Qiagen).

Molecular characterization of samples was performed by amplification and sequencing of 3 different gene loci of *T. p. pertenu*e identified during whole genome comparisons (primers used are listed in the Table S1). The PCR amplification was performed using nested-PCR protocol and the PCR products of the second step were sequenced by dideoxyterminator method [1].

Allelic profiles based on sequences of TP0548, TP0488 and TP0858 genes were assigned to individual samples. The targets, all of which are putative or bona fide outer membrane proteins, each contain small (300–600 nt) readily amplifiable regions with sequence heterogeneity among strains. The adopted nomenclature for different *T. p. pertenu*e strain types is expressed as one letter, representing tp0548 types, followed by two numbers, representing tp0488 and tp0858 types.

Altogether, three different allelic profiles were discovered: J11, S22 and T13 (corresponding to JG8, SE7 and TD6 described previously) [2]). The presence of point mutations in the 23S rRNA genes causing macrolide resistance (A2058G and A2059G) were identified by sequencing. All samples were screened by qPCR targeting *H. ducreyi* as described previously [3].

To determine genetic diversity of *T. p. pertenue* isolates at each timepoint we estimated the Mean Evolutionary Diversity by calculating the number of base substitutions per site across the concatamers of the 3 loci from each round using a Kimura 2 parameter (K80/K2P) model using the dist.dna function from ape v5.4.1 [4]. We evaluated differences between baseline diversity and later timepoints using AMOVA (1000 permutations), implemented in the pegas v0.13 [5] package in R v3.6.0 [6]. Sample distributions were plotted using ggplot2 v3.3.3 [7].

Latent yaws surveys

A 10 µL of capillary blood was obtained from all randomly selected children aged 1-15 years to perform a rapid quantitative serological test (dual-path platform [DPP] syphilis screen and confirm assay; Chembio Diagnostics, Medford, NY, USA) [8]. Naked eye and optical density microreader DPP values were recorded. A participant was considered to have positive yaws serology if the density of the treponemal line was greater than 12 (as established by the manufacturer) and the density of the non-treponemal line was ≥30 (equivalent to rapid plasma reagin [RPR] ≥1:4) [9] . A participant was considered to have high-titer serology if the non-treponemal line had a value ≥90 (equivalent to RPR ≥1:16).

Table S1. Primers used for the nested-PCR amplification of typing loci and the 23S rRNA gene of *T. p. pertenue.*

Locus	External primers (5-3)	Coordinates[1]	Length of PCR product	Source/reference	Internal primers (5-3)	Coordinates[1]	Length of PCR product	Source
TP0548	TGGGGCACTAAACCGGAAGA	593076-593095	1613 bp	[10]	GCGGTCCCTATGATATCGTGT	593225-593283	1059 bp	[11]
	TACGGGCATTTGCGGATAGG	594669-594688			GAGCCACTTCAGCCCTACTG	594264-594283		
TP0488	CGGAGCTTTTTCCCGTAA	522649-523666	1268 bp	This study	TGGGTGAAGGGTCTTGTGAC	522711-522730	1157 bp	This study
	AGCAAAGCGAATCTTCTCCA	523897-523916			GATACCTCGTCCCCTCCAAC	523848-523867		
TP0858	CTGCTCGGACGCAAGTAAAG	935876-935895	1201 bp	This study	CATTACAATGGGCACCATGA	936024-936043	1016 bp	This study
	ACTCCCACTCGCATGTTAGC	937057-937076			AGCTCGAACTCAAGCTCAGG	937020-937039		
23S rDNA[2]	CGAAGGGAAGCAGGTGTAGT	234316-234335, 282728-282747	1666 and 1658 bp	[1,12]	GTACCGCAAACCGACACAG	234380-234398, 282792-282810	629 bp	[12]
	GCGCGAACACCTCTTTTTAC	235962-235981			AGTCAAACCGCCCACCTAC	234990-235008, 283402-283420		
	GAACCGTCCCTGAAAACTCA	284366-284385						

[1]According to the Samoa D genome (CP002374.1).

[2]Both copies of 23S rDNA gene were amplified.

ADDITIONAL RESULTS

Table S2. Characteristics of participants at baseline, 6-, 12-, and 18-month surveys.

		Control (N=30438)	Experimental (N=26238)
**Baseline survey**		22033	20331
**Gender**	Female	10556 (47.9)	9755 (48.0)
	Male	11477 (52.1)	10576 (52.0)
**Age**	≤5y	3604 (16.4)	3393 (16.7)
	6-10y	2763 (12.5)	2585 (12.7)
	11-15y	3209 (14.6)	2866 (14.1)
	>15y	12457 (56.5)	11487 (56.5)
**6-month survey**		19984	16825
**Gender**	Female	9372 (46.9)	8152 (48.5)
	Male	10612 (53.1)	8673 (51.5)
**Age**	≤5y	2005 (10.0)	1781 (10.6)
	6-10y	2103 (10.5)	1863 (11.1)
	11-15y	3984 (19.9)	3491 (20.7)
	>15y	11892 (59.5)	9690 (57.6)
**12-month survey**		25791	22694
**Gender**	Female	12499 (48.5)	10890 (48.0)
	Male	13292 (51.5)	11804 (52.0)
**Age**	≤5y	4023 (15.6)	3728 (16.4)
	6-10y	3382 (13.1)	2664 (11.7)
	11-15y	4344 (16.8)	3131 (13.8)
	>15y	14042 (54.4)	13171 (58.0)
**18-month survey**		29954	25987
**Gender**	Female	14665 (49.0)	12825 (49.4)
	Male	15289 (51.0)	13162 (50.6)
**Age**	≤5y	5455 (18.2)	4127 (15.9)
	6-10y	4328 (14.4)	3655 (14.1)
	11-15y	4566 (15.2)	4451 (17.1)
	>15y	15605 (52.1)	13754 (52.9)

Figure S1. Prevalence of ulcers from each source over time.

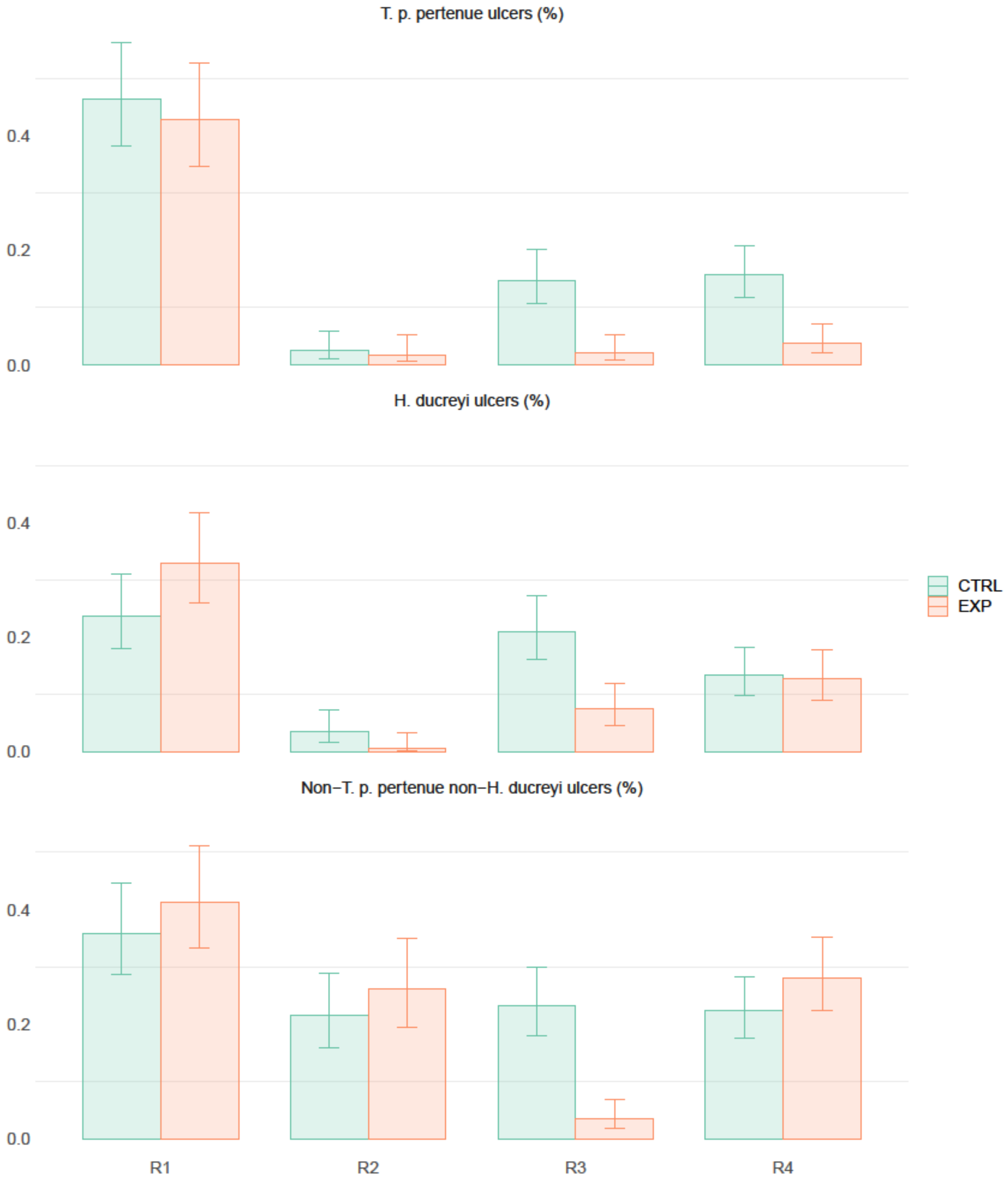

Results are presented as population prevalence with error bars showing the 95% confidence interval.

Figure S2. Change in the prevalence by age of (A) *Treponema pallidum pertenue* ulcers; (B) *Haemophilus ducreyi* ulcers, and (C) non-*T. p. pertenue* non- *H. ducreyi* ulcers.

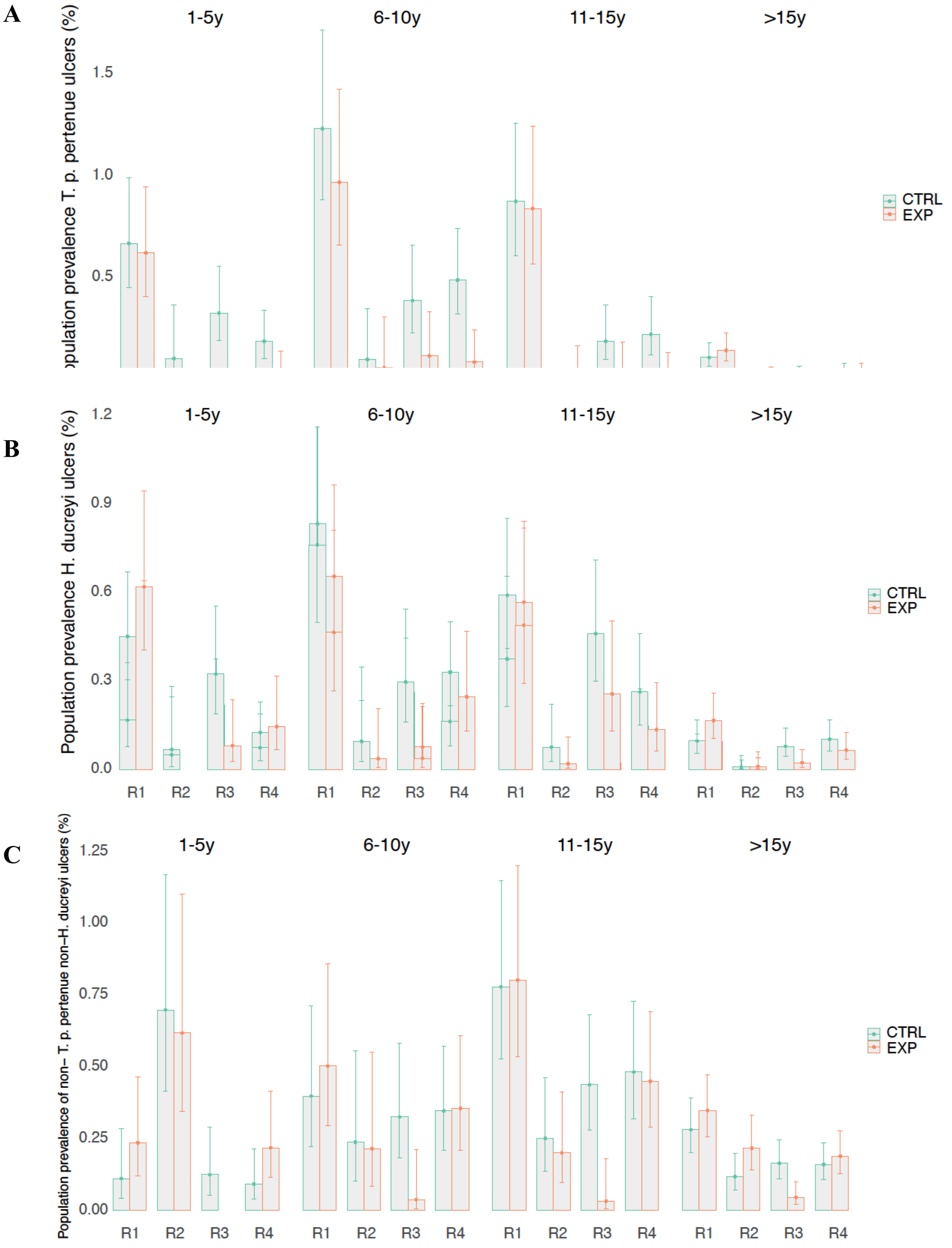

Results are presented as population prevalence with error bars showing the 95% confidence interval.

Table S3. Clinical and laboratory characteristics of PCR-confirmed yaws ulcer episodes.

	Baseline		6 months		12 months		18 months	
	Control	Experimental	Control	Experimental	Control	Experimental	Control	Experimental
**Local level government (N)**	102	87	5	3	38	5	47	10
Matalai, n (%)	26 (25.5)	11 (12.6)	0 (0.0)	3 (100.0)	10 (26.3)	2 (40.0)	7 (14.9)	5 (50.0)
Namatanai, n (%)	36 (35.3)	52 (59.8)	5 (100.0)	0 (0.0)	8 (21.1)	3 (60.0)	17 (36.2)	3 (30.0)
Sentral Niu Ailan, n (%)	40 (39.2)	24 (27.6)	0 (0.0)	0 (0.0)	20 (52.6)	0 (0.0)	23 (48.9)	2 (20.0)
**Demographics and clinical history (N)**	102	87	5	3	38	5	47	10
Age (years), *median [IQR]*	9 [6, 13]	10 [6, 14]	7 [5, 8]	11 [9.5, 18]	7 [4, 12]	8.50 [8, 10]	9 [6, 12]	12 [8, 19]
Male, *n (%)*	59 (58.4)	48 (55.2)	5 (100.0)	1 (33.3)	17 (47.2)	1 (20.0)	29 (64.4)	6 (66.7)
Ulcer duration (days), *median [IQR]*	28 [21, 84]	28 [17.5, 56]	14 [2, 14]	3 [2, 3.5]	30 [14, 60]	28 [14, 42]	28 [14, 30]	36.5 [21, 60]
Number of ulcers, *median [IQR]*	1 [1, 1]	1 [1, 2]	1 [1, 1]	2 [1.5, 2.5]	1 [1, 1]	1 [1, 1]	1 [1, 1]	1 [1, 1]
Size of ulcer (cm), *median [IQR]*	2 [1.5, 2.5]	2 [1.5, 2.5]	1.8 [1.4, 2]	4 [4, 4]	1.8 [1.2, 2.4]	2 [1, 3]	2 [1.6, 2.2]	1.6 [1.5, 2]
Immigration or travel history, *n (%)*	1 (1.0)	2 (2.7)	1 (20.0)	0 (0.0)	0 (0.0)	0 (0.0)	2 (4.8)	0 (0.0)
**Serological results (N)**	98	86	5	2	28	4	20	7
T-line ≥12, *n (%)*	73 (74.5)	72 (83.7)	4 (80.0)	2 (100.0)	23 (82.1)	4 (100.0)	11 (55.0)	5 (71.4)
T-line ≥ 12 and NT-line ≥30, *n (%)*	70 (71.4)	64 (74.4)	4 (80.0)	1 (50.0)	21 (75.0)	4 (100.00)	9 (45.0)	5 (71.4)
T-line ≥12 and NT-line ≥90, *n (%)*	51 (52.0)	45 (52.3)	3 (60.0)	1 (50.0)	12 (42.9)	3 (75.0)	5 (25.0)	4 (57.1)
**Allelic profile (N)**	82	75	3	3	31	4	39	8
J11, *n (%)*	75 (91.5)	69 (92.0)	3 (100.0)	3 (100.0)	29 (93.5)	4 (100.0)	38 (97.4)	8 (100.0)
S22, *n (%)*	3 (3.7)	6 (8.0)	0 (0.0)	0 (0.0)	1 (3.2)	0 (0.0)	1 (2.6)	0 (0.0)
T13, *n (%)*	5 (6.1)*	0 (0.0)	0 (0.0)	0 (0.0)	1 (3.2)	0 (0.0)	0 (0.0)	0 (0.0)
**23S RNA (N)**	87	81	4	1	33	5	46	10
Wild type, *n (%)*	87 (100.0)	81 (100.0)	4 (100.0)	1 (100.0)	33 (100.0)	5 (100.0)	46 (100.0)	7 (70.0)
resistant, *n (%)*	0 (0.0)	0 (0.0)	0 (0.0)	0 (0.0)	0 (0.0)	0 (0.0)	0 (0.0)	3 (30.0)

IQR: interquartile range (25th and 75th percentiles).
*A sample was coinfected with two allelic profiles (J11 and T13)

Table S4. Characteristics of individuals included in the serosurvey of latent yaws at 18 months.

	Control arm (N=994)	Experimental arm (N=945)	p-value*
**Local level government, n (%)**			
Matalai	183 (18.4)	183 (19.4)	
Namatanai	453 (45.6)	425 (45.0)	0.865
Sentral Niu Ailan	358 (36.0)	337 (35.7)	
**Age, mean (Sd)**	9.5 (2.9)	9.9 (2.8)	0.003
**Age group, n (%)**			
1-5y	96 (9.7)	64 (6.8)	
6-10y	513 (51.6)	449 (47.5)	0.002
11-15y	378 (38.0)	425 (45.0)	
Missing data	7 (0.7)	7 (0.7)	
**Gender, n (%)**			
Female	489 (49.2)	461 (48.8)	0.873
Male	505 (50.8)	484 (51.2)	

REFERENCES

1. Grillová L, Bawa T, Mikalová L, Gayet-Ageron A, Nieselt K, Strouhal M, et al. Molecular characterization of Treponema pallidum subsp. pallidum in Switzerland and France with a new multilocus sequence typing scheme. PLoS One. 2018;13(7):e0200773.
2. Godornes C, Giacani L, Barry AE, Mitja O, Lukehart SA. Development of a multilocus sequence typing (MLST) scheme for Treponema pallidum subsp. pertenue: application to yaws in Lihir Island, Papua New Guinea. PLoS Negl Trop Dis. 2017;11(12):e0006113.
3. Orle KA, Gates CA, Martin DH, Body BA, Weiss JB. Simultaneous PCR detection of Haemophilus ducreyi, Treponema pallidum, and herpes simplex virus types 1 and 2 from genital ulcers. J Clin Microbiol. 1996;34(1):49–54.
4. Paradis E, Schliep K. ape 5.0: an environment for modern phylogenetics and evolutionary analyses in R. Bioinformatics. 2019;35(3):526–8.
5. Paradis E. pegas: an R package for population genetics with an integrated–modular approach. Bioinformatics. 2010;26(3):419–20.
6. R Core Team. R Foundation for Statistical Computing. A language and environment for statistical computing. [Internet]. 2015 [cited 2021 May 23]. Available from: http://www.r-project.org/
7. Wickham H. ggplot2. Wiley Interdiscip Rev Comput Stat. 2011;3(2):180–5.
8. Marks M, Yin Y-P, Chen X-S, Castro A, Causer L, Guy R, et al. Metaanalysis of the performance of a combined treponemal and nontreponemal rapid diagnostic test for syphilis and yaws. Clin Infect Dis. 2016;63(5):627–33.
9. Ayove T, Houniei W, Wangnapi R, Bieb S V., Kazadi W, Luke LN, et al. Sensitivity and specificity of a rapid point-of-care test for active yaws: A comparative study. Lancet Glob Heal. 2014 Jul 1;2(7):e415–21.
10. Matějková P, Flasarova M, Zakoucka H, Bořek M, Křemenová S, Arenberger P, et al. Macrolide treatment failure in a case of secondary syphilis: a novel A2059G mutation in the 23S rRNA gene of Treponema pallidum subsp. pallidum. J Med Microbiol. 2009;58(6):832–6.
11. Woznicová V, Šmajs D, Wechsler D, Matejková P, Flasarová M. Detection of Treponema pallidum subsp. pallidum from skin lesions, serum, and cerebrospinal fluid in an infant with congenital syphilis after clindamycin treatment of the mother during pregnancy. J Clin Microbiol. 2007;45(2):659–61.
12. Lukehart SA, Godornes C, Molini BJ, Sonnett P, Hopkins S, Mulcahy F, et al. Macrolide resistance in Treponema pallidum in the United States and Ireland. N Engl J Med. 2004;351(2):154–8.
